# 12-month prevalence of atopic dermatitis in resource-rich countries: a systematic review and meta-analysis

**DOI:** 10.1038/s41598-022-19508-7

**Published:** 2022-09-06

**Authors:** Annika Volke, Karolin Toompere, Kaja-Triin Laisaar, Marje Oona, Anna Tisler, Annika Johannson, Kadi Kallavus, Katrin Lang, Ele Kiisk, Anneli Uusküla

**Affiliations:** 1 Department of Dermatology, Institute of Clinical Medicine, University of Tartu, Raja 31, 50417 Tartu, Estonia; 2 Dermatology Clinic, Tartu University Hospital, Tartu, Estonia; 3 Institute of Family Medicine and Public Health, University of Tartu, Tartu, Estonia; 4 Diagnostic Service, Pärnu Hospital, Pärnu, Estonia

**Keywords:** Epidemiology, Skin diseases

## Abstract

There is a lack of robust prevalence estimates of atopic dermatitis (AD) globally and trends over time due to wide variation of populations and age groups studied, different study methodologies and case definitions used. We sought to characterize 12-month AD prevalence across the life span and change over time in resource-rich countries focusing on population-based studies and using a standardized AD case definition. This systematic review was conducted according to PRISMA guidelines. Medline (Ovid), Embase, WOS core collection, Cinahl, and Popline were searched for studies published since inception through August 15, 2016. Studies were synthesized using random effects meta-analysis. Sources of heterogeneity were investigated using subgroup analyses and meta-regression. From 12,530 records identified, 45 studies met the inclusion criteria. Meta-analysis with random effects revealed the 12-month period prevalence of 9.2% (95% confidence interval 8.4–10.1%). The prevalence was significantly higher among 0–5-year-old children (16.2%; 95% confidence interval 14.2–18.7%) than in older age groups. Studies using a random sampling strategy yielded lower prevalence estimates than studies relying on other sampling methods. There was no clear time trend in AD prevalence over the period of 1992–2013.

## Introduction

Atopic dermatitis (AD) is a common inflammatory skin disease that has been shown to mount a substantial psychological, social and economic charge to patients, their families and society^1–4^. The clinical concept of AD encompasses a wide spectrum of phenotypes regarding clinical features, severity, course, patient’s age and ethnicity as well as the development of comorbid disease and response to treatment^5,6^. Several genetic, immunologic and environmental factors contribute to the complex pathophysiology of AD, but the key driver is a subject of debate^7,8^.

The onset of AD is usually in early childhood, but the natural course of the disease has not been easy to predict^9–13^. Perhaps not surprisingly, most epidemiological studies have been conducted in children although nowadays it is increasingly recognized to persist into, or to begin also in, adulthood or even the elderly^14–18^.

In high-income countries, AD is considered one of the most common cutaneous inflammatory disorders^3,4,7^. Yet the studies on AD prevalence have been difficult to interpret because they differ in methodology—in terms of disease definition, sampling frame and methods, regions, or age groups^19,20^. Partly arising from the same methodological problems, literature is remarkably scant on time trends. We retrieved two studies estimating worldwide time trends of AD prevalence. In a systematic review of sequential data Deckers at al.^20^ found that the prevalence of AD was increasing in some regions with no clear trends in others. Although the analysis was restricted to studies with validated instruments only, the authors admitted that assessing trends was complicated by the wide range of outcome measures and changes in diagnostic criteria over time. Another report of secular trends brought forth that AD prevalence was plateauing in some populations with previously high prevalence rates while still increasing in others^21^. The study engaged only children and data were drawn from two identically designed phases of a large international multi-site study. Thereby, there is a lack of global robust prevalence estimates of AD in all ages and trends over time.

The objective of this study was to systematically review research on 12-month AD prevalence in the general population of resource-rich countries. Unlike Deckers et al^20^ we limited our search to population-based surveys and accepted somewhat wider criteria of explicit AD. In addition, we explored variations in prevalence based on age, gender, period, study design, region, and AD case definition.

## Material and methods

We systematically reviewed population-based studies from EU/EEA and other high-income countries published between 1991 and 2016 where AD prevalence in the previous 12 months was presented or could be calculated from available data. We followed the Preferred Reporting Items for Systematic Reviews and Meta-Analyses guidelines^22^.

### Eligibility criteria

To be included, studies had to be published in English and report original research. Eligible study designs were cross-sectional surveys or baseline evaluation in cohort studies that used population-based sampling methods and assessed 12-month period prevalence of AD. Studies with the following characteristics were excluded: narrative and systematic reviews; case control studies; birth cohort studies; experimental studies; and data published in letters, commentaries, and editorials.

We limited our inclusion to nationally representative samples of general population. We considered a well-defined general population based sampling strategy would apply to population-based studies^23^. Such studies encompass those that are defined by national and sub-national geographic boundaries of a country (including school or kindergarten if the sampling frame included all the institutions in the region) as well those defined by membership in health maintenance organizations^23^. We focused on AD prevalence in resource-rich countries from the EU/EEA^24^, and non-European high-income countries (as defined by the Organization for Economic Cooperation and Development^25^). Studies where the AD diagnosing criteria did not match our AD case definition (see below) were excluded from this review.

### Information sources and search strategy

We searched Medline (Ovid), Embase, WOS core collection, Cinahl, and Popline from their inception until 15th August 2016. Search strategies, adapted for each search engine, included terms for “atopic dermatitis”, “atopic eczema” and “prevalence” and individual names of EU/EEA countries (and Switzerland), or “Europe”, or the non-European high-income countries (Australia, Canada, Chile, Israel, Japan, Korea, New Zealand, USA) (see Supplementary material 1). In addition, we searched reference lists for eligible studies. The full electronic search strategy for Medline (Ovid) is presented in Supplementary table 1.

### Selection process

Pairs of qualified reviewers independently screened the titles and abstracts, then by full text to determine eligibility for final inclusion (following the predefined inclusion criteria) and recorded the results onto standardized forms of a preformatted data collection template. At both stages of screening, any differences between reviewers were discussed and a consensus decision for eligibility and inclusion was made for all articles; a third reviewer resolved differences between reviewers if necessary.

### Data collection process

A data extraction sheet was developed based on the Cochrane Consumers and Communication Review Group's data extraction template^26^, pilot-tested on eight randomly selected but included studies and refined accordingly. If multiple publications reported one study, we extracted data from the primary publication (assigned as the publication with the most detailed description of the methods and the most data on specified prevalence measures). Data reported in the primary publication were used in case of inconsistencies between the publications based on a same source study. The two reviewers compared the extracted data and resolved differences by discussion. If there was still a discrepancy, a third reviewer adjudicated. We did not contact authors for additional information.

### Data items

The following information was extracted: study design; country; setting (national or sub-national); demographic characteristics (age, gender); sampling method (random—simple, stratified, multi-staged, cluster; or convenience sampling); numbers of eligible, invited and participating subjects; number of subjects excluded and those with detected AD; definition of AD used; estimated prevalence and 95% confidence intervals (CI) reported in the study.

### Study risk of bias assessment

We used published guidelines for cross-sectional prevalence studies by Boyle^27^ to assess the risk of bias related to methodological aspects of included studies and disagreement at any stage was solved by consensus or arbitration. The items assessed included: representativeness of the target and source populations; similarity of responders and non-responders; attained sample size; use of standardized/valid AD measurement/definition; appropriateness of statistical methods; and response rate. We pre-specified criteria to determine whether each of the features in a specific study could be rated as attributing a low or high risk of bias, or if there was insufficient information to decide (unclear). The overall risk of bias generally corresponded to the highest risk of bias in any of the items. However, if a study was judged to have ‘unclear’ risk of bias for multiple (two or more) items, it was regarded as at high risk of bias overall. Publication bias was assessed qualitatively using funnel plot symmetry as a surrogate for low risk of publication bias.

### Data analysis and synthesis

We estimated the 12-month AD prevalence using the number of individuals with AD and the number of people tested (confidence intervals (CI) are based on the Clopper-Pearson method). Where authors reported stratified sampling methods, the published point estimate and 95% CI were used. Where simple random sampling has been used and data were available, we calculated AD prevalence with binomial 95% CI. We examined time trends in the AD prevalence estimates using meta-regression regression models with the prevalence estimates as the outcome variable and the midpoint of the data collection years as the predictor.

We calculated a response rate for each study using an algorithm to define numerators and denominators consistent with the recommendations of the American Association for Public Opinion Research^28^. Where available, the numerator was the number of people having AD and the denominator was the number of eligible subjects asked to participate, able to participate, or sent an invitation for study participation. If the study report did not include these figures, we used the number of people studied, followed by the number of study subjects used in the analysis as the numerator and the number of eligible people as the denominator. We used the published response rate in studies that used complex sampling methods and post-stratification weighting.

Prevalence estimates were pooled using random effects meta-analysis (generalized linear mixed model) to derive the average of the study estimates and their 95% CI, as suggested by Schwarzer et al.^29^. We assessed statistical heterogeneity using the Q-test and *I*^2^ statistic. The *I*^2^ statistic was interpreted according to the recommended thresholds^30^. Sensitivity analysis was undertaken to explore whether the results were sensitive to restriction of studies with low risk of bias.

Clinical and methodological heterogeneity was assessed in subgroup analysis using meta-regression. Variables for subgroup analysis were selected a priori: age (when study source population could be designated into age groups of 0–5, 6–12, 13–18 or over 18 years); geographic coverage (Asia versus elsewhere); study response rate (< 70%,70–79%, 80% +); time period of data collection (1991–2000, 2001–2010, 2011–2016); population setting (health care institution, elsewhere); sampling (simple random sampling, other); type of AD measure (patient self-report of symptoms; self-report of AD diagnosed by a doctor; other). Calculations were performed with R function metaprop from package meta^31^.

### Outcomes of the study

The primary outcome was 12-month prevalence of AD. Secondary outcomes included the prevalence of AD across age, sex, study decade, AD case definition and country/region. For the current review, AD was pre-defined as (1) an itchy skin condition with a chronic and/or relapsing course and affecting the folds of the elbows, behind the knees, in front of the ankles, under the buttocks, or around the neck, ears or eyes^32,33^, or (2) diagnosed by a physician: (i) based on the self/parent report on atopic dermatitis/eczema diagnosis; (ii) observed in the study; or, (iii) extracted from a healthcare maintenance/administrative database according to the International Statistical Classification of Diseases and Related Health Problems (ICD-9/10) AD-specific diagnosis codes. Institutional review board approval was not sought as only data from already published studies was used.

## Results

**Figure 1:**
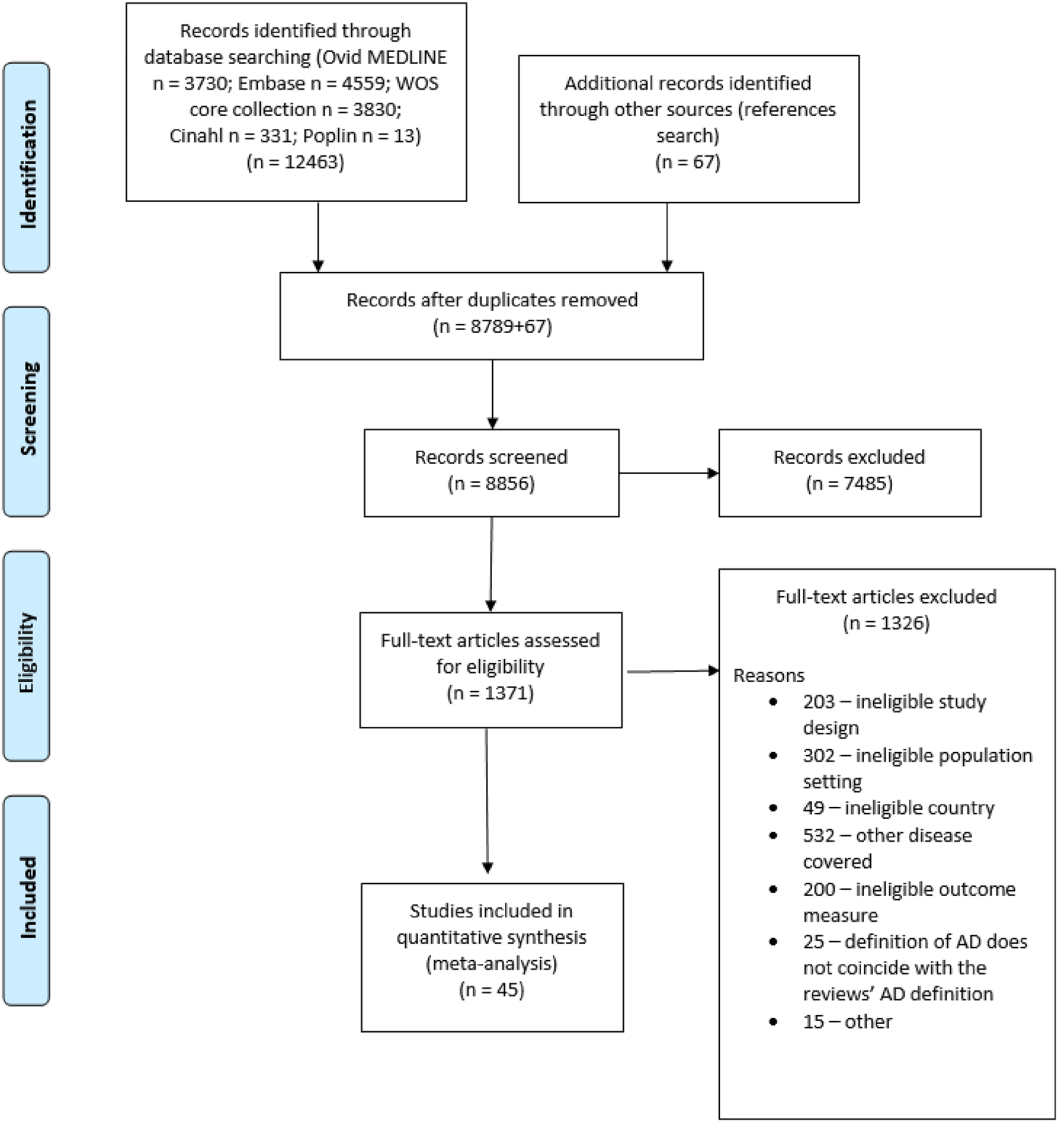
The study selection process for the systematic review and meta-analysis.

After removing duplicates, the search retrieved 8,856 records. After title and abstract screening, 7485 records were excluded. The remaining 1371 articles were read in full and screened for eligibility. After excluding 1326 articles for ineligibility, 45 studies remained for analysis. Expressly, out of the 532 studies excluded for covering other diseases, in 432 cases the disease investigated was defined as “eczema” (e.g., eczema or patient or doctor reported eczema or childhood eczema or food-sensitized and non-sensitized eczema or hand eczema) with no details allowing further clarification. In the rest of excluded studies hay fever (*n* = 39), food allergy (*n* = 28), asthma (*n* = 14), allergic rhinitis (*n* = 12) or atopy (*n* = 7) were studied. The flow chart of the selection process and reasons for excluding studies is detailed in Fig. 1.

### Included studies

The data of the 45 included studies had been collected from 27 countries between 1992 and 2013 and published during the period from 1998 to 2016. Altogether, AD prevalence was assessed in 75,203,859 individuals (3,494,054 when excluding individuals whose data was obtained from healthcare databases) (Supplementary Fig. 1). There were eight studies from South Korea^34–41^, seven from Japan^42–48^, five from Germany^49–53^, three from the UK^54–56^, two studies from Denmark^57,58^ and the USA^59,60^ and one from Italy^61^, Canada^62^, Croatia^63^, Cyprus^64^, Finland^65^, Lithuania^66^, New Zealand^67^, Poland^68^, the Netherlands^69^, Norway^70^, Spain^71^, Sweden^72^ and Switzerland^73^, respectively. Five studies reported data from multiple countries^74–78^, providing information also from France, Greece, Estonia, Latvia, Iceland, Belgium, Portugal and Hungary in addition to the aforementioned states. Table 1 summarizes the characteristics of each study.

**Table 1:** Characteristics of included studies.

Source	Country	Data collection period	Sample size	Study subjects’ age (in years)	Response rate (reported by authors)	Sampling strategy	Sampling unit	AD definition
Choi^34^	South-Korea	2008	6453	0–6	70–79	Convenience	School	ISAAC
Cibella^61^	Italy	2005–2006	2150	10–17	80 +	Unclear	School	Modified ISAAC
Duhme^49^	Germany	1994–1995	13,123	5–8; 12–15	80 +	Random	School	ISAAC
Emerson^54^	UK	1995–1996	1761	1–5	80 +	Convenience	General practice	UK
Flohr^76^	France, Greece, Italy, Netherlands, Norway, Spain, UK, Latvia, New Zealand	1998–2004	20,049	8–12	NR	Random	School	ISAAC
Flohr^75^	Greece, Norway, Spain, Latvia, Iceland, New Zealand	1998–2004	11,241	8–12	NR	Random	School	ISAAC
Flohr^74^	France, Greece, Netherlands, Norway, Spain, Latvia, Estonia, New Zealand	1998–2004	11,587	8–12	NR	Random	School	ISAAC
Garcia-Marcos^77^	Estonia, Hungary, Lithuania, Poland, New Zealand, Belgium, Portugal, Spain, Japan, Canada, USA, Finland	2001–2003	142,085	6–7; 13–14	NR	Random	School	ISAAC
Anderson^55^	UK	1995, 2002	30,838	12–14	80 +	Random	School	ISAAC
Grize 2006^73^	Switzerland	1992, 1995, 1998, 2001	5446	5–7	70 +	Convenience	School	ISAAC
Guiote-Domínguez^71^	Spain	2005	381	6–7; 13–14	NR	Random	School	ISAAC
Hong^35^	South-Korea	2008	10,383	0–13	NR	Convenience	School	ISAAC
Kudzyte^66^	Lithuania	1994–1995	1879	6–7	80 +	random	school	ISAAC
Kurosaka^42^	Japan	2005–2006	11,116	6	80 +	Convenience	School	ISAAC
Lee^36^	South-Korea	2005	8631	0–19	NR	Random	Individual	Self-report of physician diagnosis
Augustin^50^	Germany	2009	293,181	0–18	NA	Random	Health insurance data	ICD-10
Miyake^43^	Japan	2004–2005	23,338	6–15	NR	Random	School	ISAAC
Miyake^44^	Japan	2001	5539	12–15	NR	Random	School	ISAAC
Mortz^57^	Denmark	1995–1996	1501	12–16	NR	Random	School	Hanifin & Rajka
Radtke 2014^51^	Germany	2009	1,349,671	18–100	NA	Random	Health insurance data	ICD-10
Sasaki^45^	Japan	2012	28,343	6–12	NR	Unclear	Individual	ISAAC
Saunes^70^	Norway	1995–1997	8393	13–19	80 +	Random	Individual	ISAAC
Silverberg^59^	USA	2005–2006	4970	20-	70–79	Random	Individual	Modified ISAAC
Ukawa^46^	Japan	unclear	4254	6–12	< 70	Convenience	School	ISAAC
Kim^72^	Sweden	2000, 2008	17,946	15	< 70	Random	Individual	ISAAC
Asher^67^	New Zealand	1992–1993	31,083	6–7; 13–14	80 +	Random	School	ISAAC
Asher^78^	UK, Spain, Canada	2002–2003	67,414	6–7; 13–14	NR	Random	School	ISAAC
Kolokotroni^64^	Cypros	1999–2000; 2007–2008	7160	7–8	80 +	Random	School	ISAAC
Austin^56^	UK	1995	27,507	12; 13; 14	80 +	Random	School	ISAAC
Lee^37^	South-Korea	2008	8644	6–11; 12–14	80 +	Random	School	Modified ISAAC
Lee^38^	South-Korea	2012–2013	1820	6–12; 12–15; 15–18	NR	Random	School	Modified ISAAC
Banac^63^	Croatia	2001–2002, 2009–2010	6060	6–7; 13–14	80 + ; 70–79	Random	School	ISAAC
Oh^41^	South-Korea	1995; 2000	82,631	6–12; 12–15	80 +	Random	School	ISAAC
Remes^65^	Finland	1994–1995	11,607	13–14	80 +	Random	School	ISAAC
Silverberg^60^	USA	2005–2006	3049	8–11; 12–15; 16–19	80 +	Random	Individual	Modified ISAAC
Sugiyama^47^	Japan	1995–1996	4466	13–14	NR	Unclear	School	ISAAC
Sybilski^68^	Poland	2006–2008	18,617	6–7; 12–14; 20–44	< 70	Random	Individual	ISAAC
van de Ven^69^	Netherlands	2003	9713	12; 13; 14	80 +	Random	School	modified ISAAC
Wang^62^	Canada	2003	8334	13–14	NR	Random	School	ISAAC
Yura^48^	Japan	1993–2006	2,802,403	7–12	80 +	Random	School	Self-report of physician diagnosis
Zutavern^52^	Germany	1995–1996	11,904	5–11	70–79	Random	School	modified ISAAC
Worm^53^	Germany	1998–2000	1739	18–65	< 70	Random	Individual	Hanifin & Rajka
Vinding^58^	Denmark	2010–2013	16,507	30–39; 40–49; 50–59; 60–69; 70–79; 80–89	< 70	Random	Individual	Self-report by modified UK diagnostic criteria
Park^39^	South-Korea	2005	1989	20–39; 40–59; 60-	NR	Random	Individual	Self-report of physician diagnosis
Yu^40^	South-Korea	2003–2008	NA	< 2; 2–5, 6–18	NA	Random	Health insurance data	ICD-10

AD—atopic dermatitis; NR—not reported; NA—not applicable; ISAAC—criteria of the International Study of Asthma and Allergies in Childhood; Hanifin & Rajka—self-reported score of symptoms based on the Hanifin and Rajka criteria; UK—physician’s diagnosis based on UK refinement of diagnostic criteria; ICD-10—physician’s diagnosis according to ICD-10.

### Risk of bias assessment and response rate

**Figure 2:**
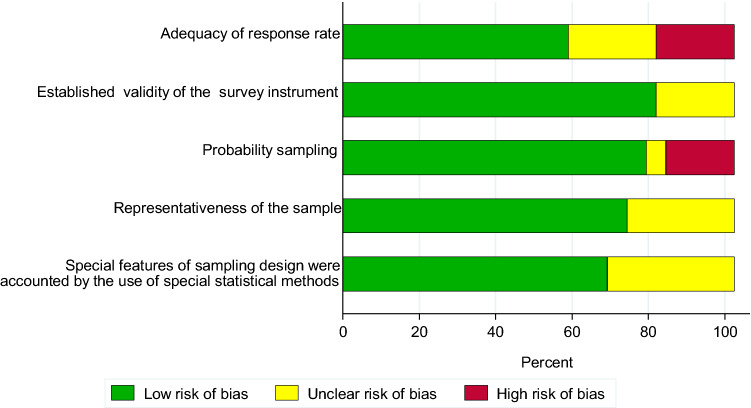
Risk of bias assessment.

The assessments of risk of bias for each included study are presented in Supplementary table 2. Given the very limited information on data collection methods and response rates provided by the five studies of multiple countries^74–78^, formal risk of bias could not be assessed in these studies. Of note is that all of these studies compiled data from an international multisite collaboration research project based on a standardized study design^32,33^. From the remaining 40 studies, twelve (27%) were considered to have high risk of bias, and in 15 (33%) the risk was unclear, at least for one item assessed (Fig. 2). Thirty six studies out of the 45 (80%) utilized random sampling, six studies convenience sampling^34,35,42,46,54,73^ and in three^45,47,61^ the sampling strategy was unclear. In 24 of the studies utilizing random sampling, the sampling unit was a school^36,37,40,42,43,47,48,52,54–56,61–66,68,70,74–78^, in five the sampling unit was at an individual level^39,53,58,70,72^, four studies used multistage sampling (regional and individual)^36,59,60,68^, and three studies were based on health insurance data^40,50,51^. The studies using convenience sampling, or for which the sampling strategy was unclear, were regarded to be at high risk of bias in relation to the sampling from the source population.

The target population was assumed to represent the general population in 30/45 (67%) of studies. However, none of the studies provided a comparison between participants and non-participants and none of the studies gave enough information about the source population to determine whether or noth this was representative of the target population. Amongst the 40 studies with some site or country specific data available, only 26 (65%) reported a response rate or data allowing estimations to be made from them. From these, 17 studies had a response rate above 80%^37,41,42,48,49,54–56,60,61,63–67,69,70^, four had a response rate of between 70 and 80%^34,52,59,63^, and five below 70%^46,53,58,68,72^. The potential for publication bias assessed through funnel plots (stratified by age group) did not suggest a significant bias (Supplementary Fig. 2).

### Measurement of AD in the studies

In most of the studies (38/45, 84%), AD case definition was based on the self-report of symptoms. Among them, in 28 studies^34,35,41–47,49,55,56,62–68,70–78^ the questionnaire of the International Study of Asthma and Allergies in Childhood (ISAAC)^32^, and in 7 studies^37,38,52,59–61,69^ modifications of the ISAAC instrument, were used. In the remaining two studies^53,57^, the self-reported score of symptoms were guided by criteria suggested by Hanifin and Rajka^79^, and in one study^58^ by modified UK diagnostic criteria^80^. In three studies^36,39,48^, a self-report of physician diagnosis of AD was used and in one study^54^ AD was diagnosed by a physician based on a UK refinement of diagnostic criteria^81^. In three of the included studies^40,50,51^ assessment was based on ICD-10 diagnostic codes for AD in administrative health data.

### 12-month prevalence of AD

**Figure 3:**
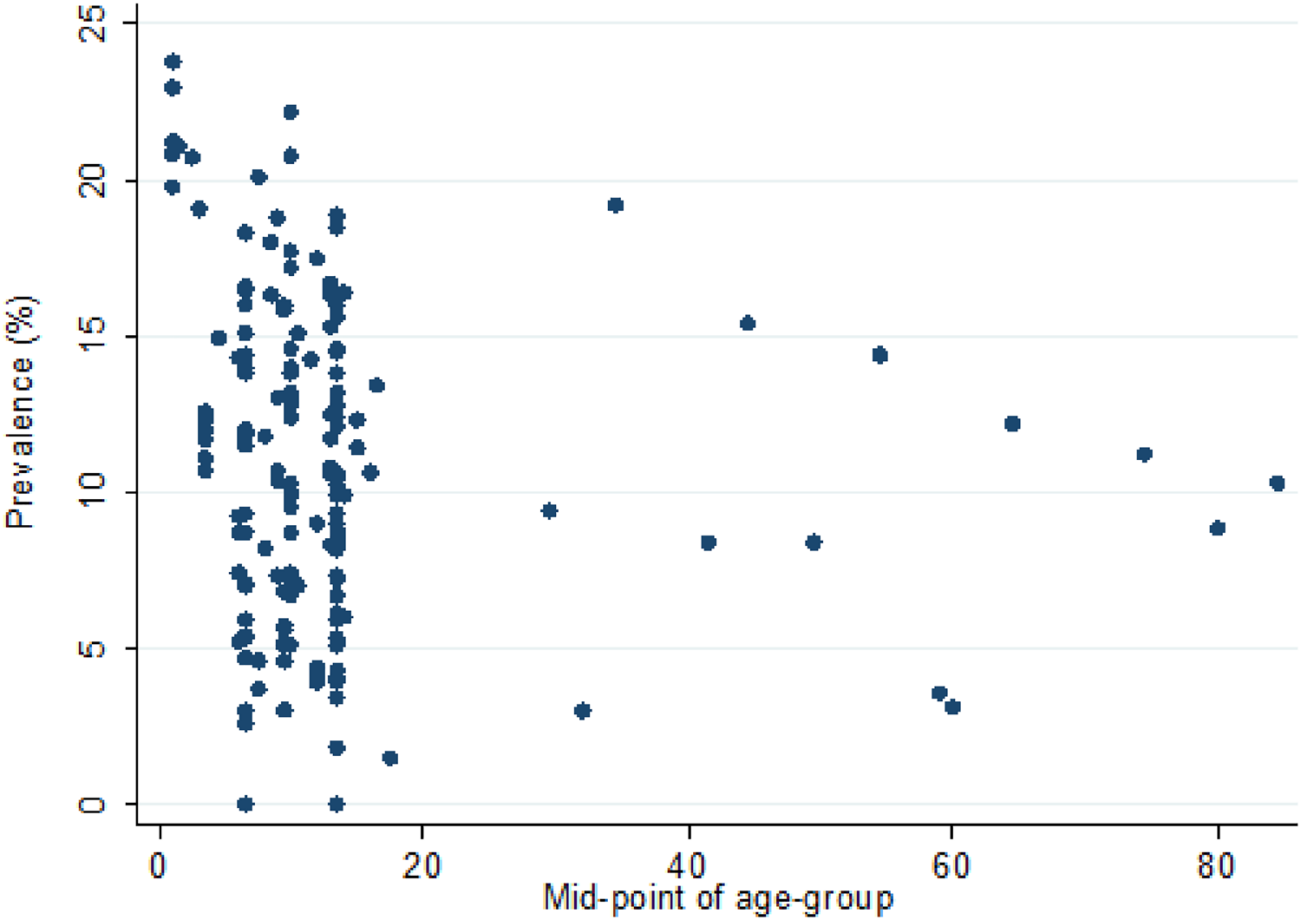
12-month prevalence of AD across age groups.

AD prevalence estimates ranged from 0 to 24% (Fig. 3). Meta-analysis identified overall 12-month period pooled prevalence of AD across all included studies of 9.2% (95% CI 8.4–10.1%) with a high level of heterogeneity. Meta-regression was used to explore potential variables that may have accounted for the observed high heterogeneity (Table 2). Female gender predicted higher AD prevalence (11.8 vs 8.2%; *p* = 0.0063). Then the analysis was stratified by age groups. Altogether, there were 17 prevalence estimates available for children aged 0 to five years (3 studies)^34,40,54^, 81 for children aged 6–12 years (21 studies)^37,38,41,42,45,46,48,56,60,61,63,64,66,68,69,71,74–78^, 41 for children aged 13–18 years (13 studies)^38,47,56,61–63,65,68,69,71,72,77,78^ and 11 prevalence estimates for adults (4 studies)^39,58,59,68^ in the included studies. Quantitative analysis yielded a pooled AD prevalence of 16.3% among children aged 0–5 years (95% CI 14.2–18.8%; 18,573,027 participants), 9.4% among 6–12-year-olds (95% CI 8.2–10.8%; 3,071,305 participants), 8.3% among 13–18-year-olds (95% CI 6.6–10.4%; 222,021 participants), and 9.3% among adults (95% CI 6.6–13.0%; 32,866 participants). For the youngest age group (0–5 years), the data could be drawn from just two countries, South Korea and the UK. Sensitivity analysis of age grouping did not reveal significant bias (Supplementary table 2).

**Table 2:** Subgroup analyses of factors associated with 12-month AD prevalence.

Variable	Category	No of prevalence estimates	Mean prevalence, % (95% CI)	*p*-value
**Individual characteristics**				
Age	0–5	17	16.3 (14.2; 18.8)	Ref
	6–12	81	9.4 (8.2; 10.8)	0.0005
	13–18	41	8.3 (6.6; 10.4)	< 0.0001
	19 +	11	9.3 (6.6; 13.0)	0.0140
Gender	Female	26	11.8 (9.9; 14.0)	Ref
	Male	26	8.2 (6.8; 9.9)	0.0063
**Study characteristics**				
Sampling unit	Health insurance data	19	10.4 (7.7; 13.9)	Ref
	Pre/school	82	10.1 (9.1; 11.2)	0.8304
	Individual	22	7.7 (5.7; 10.3)	0.0847
	Other	4	17.0 (13.7; 20.8)	0.0925
Sampling	Random sampling	164	8.9 (8.0; 9.8)	0.0050
	Other	17	13.9 (11.6; 16.5)	Ref
Response rate	< 70%	19	11.0 (8.6; 13.9)	Ref
	70–79%	9	7.9 (5.7; 10.8)	0.1475
	≥ 80%	59	9.5 (8.1; 11.0)	0.3297
Outcome assessment measure	Self-reported symptoms	156	9.2 (8.3; 10.1)	Ref
	Self-reported AD^a^	10	7.0 (5.5; 8.8)	0.2066
	Other	24	10.8 (8.3; 14.1)	0.2247
**Study configuration**				
Region	Asia	53	9.0 (6.9; 11.6)	0.9724
	Non-Asia	137	9.3 (8.5; 10.1)	Ref
Study period	1991–2000	85	10.4 (9.5; 11.5)	Ref
	2001–2010	88	7.4 (6.2; 8.8)	0.0004
	2011–2016	10	14.1 (12.5; 15.9)	0.1377

^a^Diagnosed by a health care practitioner.

Further, the effect of study quality on the primary outcome was tested. Limiting the analysis to studies with low risk of bias (10 studies with 34 prevalence estimates^48–51,56,57,60,65,67,70^) gave an AD prevalence of 8.9% (95% CI 7.2–11.0). This did not differ significantly from the prevalence estimate based on all included studies. Of note is that no studies with low risk of bias were detected within populations designated to age limits of either 0–5 or over 18 years.

**Figure 4:**
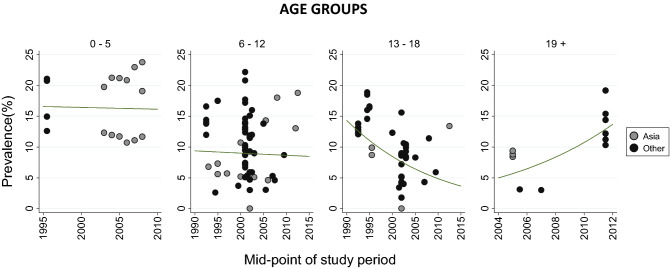
12-month prevalence of AD by age groups (0–5, 6–12, 13–18 and 19 + years) and region over time.

Besides age and gender, the methodological characteristics of studies and the period of data collection were significantly associated with AD prevalence in meta-regression. The prevalence of AD was lower in studies reporting data from 2001–2010 compared to 1991–2000 (7.4 vs. 10.4%; *p* = 0.0004). Next, we stratified the time trend analysis by age group. There was a slight decrease in 12-month prevalence among 0–5-year-old and 6–12-year-old children from mid-1990s to the late 2000s (not statistically significant, *p* = 0.9119, and *p* = 0.8259, respectively; Fig. 4). Among individuals aged 13–18 years, a significant downtrend in predicted AD prevalence was observed over the same time (*p* = 0.0122), from 12.3% (95% CI 8.5–17.4%) in 1993 to 4.3% (95% CI 2.5–7.6%) in 2012. In the adult group, we saw an increase in AD prevalence over the past decades (*p* = 0.0036), from 5.7% (95% CI 4.0–8.7%) in 2005 to 13.7% (95% CI 9.6–19.2%) in 2012 (Fig. 4).

Studies using random sampling yielded lower AD prevalence than studies of non-random sampling (8.9 vs 13.9%; *p* = 0.005). There were no differences between Asia and other regions either in overall AD prevalence (Table 2) or in trends across age groups (Fig. 4). We further explored the effects of the case definition of AD through sensitivity analysis. AD prevalence was 9.2% (95% CI 8.3–10.1%) in studies using self-report on symptoms, 7.0% (95% CI 5.5–8.8%) in studies using self-report on physician diagnosis (*p* = 0.2066) and 10.8% (95% CI 8.7–14.9%) in studies using other measurement methods (*p* = 0.2247).

## Discussion

Our study describes the prevalence and trends of AD over the past three decades in resource-rich countries. Drawn from pooled data from countries of Europe, North America, East Asia and Oceania, we have come to two main findings. Firstly, it ascertains that nearly one-tenth (9.2%) of all people have experienced AD during last 12 months. Secondly, the prevalence of the disease has remained stable during the last decades.

We saw the highest 12-month AD prevalence of 16.3% in the youngest age group (0–5 years old), being almost twice as high as in older age groups. Our finding of higher AD prevalence among the youngest children corroborates previously published evidence that AD occurs more frequently in early life^3,4,7,82^. Also, longitudinal cohort studies revealing distinct disease trajectories in childhood and adolescence have depicted that the most prevalent subphenotypes are the ‘early-onset-early-resolving’ ones^9,10^.

In our analysis, the prevalence of AD in children aged 6–18 years and adults did not differ. This is concordant with the finding from a systematic review of longitudinal studies from Northern European countries of similar AD prevalence in the age groups of up to 12 years and older^83^. Likewise, in two British birth cohorts with a longer follow-up period, the annual period prevalence of AD at the age of 5 years and onwards ranged from 5 to 14% with no clear trend across ages^16^. The steady prevalence across ages older than 5 years probably reflects a balance of different disease trajectories, i.e., persistent disease as well as the phenotypes of AD that have resolved or relapsed for that period and later-onset disease. The observed 12-month prevalence of 9.3% among adults is echoed in the latest study from Finland where the prevalence an AD of 10.1% in the adult population was detected^84^.

Previous studies on gender differences in AD prevalence have come to conflicting results^85^. We found the overall female preponderance of 1.4:1.0. This finding is in agreement with the recent systematic review documenting a higher burden of AD among women throughout all age groups and geographic regions^3^. It has been speculated that skin care practices, occupational exposures, higher awareness or disease misclassification can play a role in this phenomenon, but to our knowledge these factors have not been formally studied. In a recent analysis, AD was not associated with endogenous sex hormones, neither in adolescents nor adults^86^.

Although there was a transient decrease of reported AD prevalence in the period 2001–2010, no convincing time trend was disclosed across the three decades. This observation is in line with the findings of the collaborative research looking at the secular trends in childhood AD and documenting the levelling off or decreasing prevalence of AD in some formerly high prevalence sites from high-income countries^21^. The nature of current study precludes assessment of causes of the drop in AD prevalence witnessed among 13–18-year-olds over time. Neither does it discriminate whether children born at late 1980s and early 1990s were less likely to have persistent AD or to develop AD later at school age, or both. So far, no age-group specific individual or environmental risk factors have been identified in the pathogenesis of AD^87^. Across studies, the most important predictors of AD persistence beyond childhood have been earlier age of onset, disease severity, allergic multimorbidity, family history of atopy, filaggrin gene mutations, and urban environment^88^. None of these appear to be modified easily. We propose that there could be a cohort effect attributable to not yet known changes in exposome since 1990s (e.g., more stringent use of antibiotics or promotion of breastfeeding). Another way to explain the decline would be more efficient treatment of AD (e.g., liberal use of emollients or proactive therapy) leading to changes in disease course. Interventional or prospective cohort studies of longer duration than usual are needed to clarify this. Concomitantly, we saw some increase of AD prevalence among people aged 19 years and older over time. One can speculate that there is no real downtrend of prevalence in adolescents, but the manifestation of AD is postponed into adulthood due to environmental or behavioural changes. Nevertheless, considering that late-onset AD was largely ignored until the 1990s^14,89^, it cannot be excluded that the increase of AD prevalence found in this age group is due simply to a rise in awareness of AD in adults. It has been debated whether anyone could develop clinical syndrome of AD if exposed to enough key risk factors or a finite number of only genetically predisposed individuals are susceptible to it^90,91^. On a large scale, stable AD prevalence throughout time and ages gives support to the latter hypothesis and reassures that the epidemic is not increasing infinitely.

In our review, the sampling method was the only study design item associated with AD prevalence. Most of the included studies used a random sampling strategy and the remainder were mainly based on convenience samples. The latter yielded significantly higher prevalence estimates. This could have been anticipated since non-probability sampling based studies are known to have disadvantages and often oversample individuals with the condition studied due to overrepresentation either by respondents’ self-selection, membership bias or non-responding^92–94^.

To-date, there is no consensus on how to capture AD at population level. Without a pathognomonic biomarker available, history taking and clinical signs are needed for diagnosis of AD and a clinician's assessment is considered the gold standard^7,95–97^. The fluctuating course of the disease further complicates collecting reliable data in population settings^91^. Previously, higher prevalence of AD has been reported in studies using participants self-report of AD compared to health care practitioner’s assessment^98^. Interestingly, in the current analysis, we saw no difference in AD prevalence whether the outcome was measured by the diagnosis made by a health care practitioner or by a participant’s self-report. Likewise, we did not reveal any differences across Asia vs other regions that have been described in some previous studies^3,12^. Despite the regional variations in single features of AD^99^ our results indicate that the entity of AD might be the same in the setting of resource-rich countries. Thus, a standardized set of self-reported items delineating pruritic, inflammatory skin condition, characterized by a chronic and relapsing dermatitis in typical anatomical sites^100^ could reliably detect AD in population-based research.

There are several strengths to this review. To our knowledge, this is the largest systematic review of AD prevalence with a comprehensive literature search, succinct focus on source studies’ design validity (population-based studies, delineating sampling strategies) and using a pre-defined standardized AD definition as an inclusion criterion. Importantly, research findings may differ substantially if different definitions of AD are used^19,101^. In our study, despite within-sample heterogeneity, the AD prevalence estimates were quite similar irrespective of the ascertainment method.

There are also limitations that should be considered in the appraisal of the evidence presented by this review. High heterogeneity was observed between studies both overall and across subgroups. We explored whether study design aspects, geographic variation, or study period could explain part of the heterogeneity. However, the remitting and relapsing nature of the disease and residual study-level differences could have contributed to unexplained heterogeneity in outcome estimates. Namely, source population and sampling characteristics were often not described in enough detail and most studies using multistage sampling disregarded the complex design when estimating AD prevalence. High heterogeneity may affect interpretation and generalization of the results. Secondly, high-income countries included in the current study were defined by their EU/EEA and/or OECD status. This has closed out data from some other high-income countries such as Singapore and Taiwan. Thirdly, as we aimed to involve only data from affluent countries, the results cannot be attributed directly to low-income regions of the world. And last, but not least, in the analysis we were able to reckon only with the factors available in the original studies. Therefore, it was not possible to assess the effect of risk factors, such as living environment, migration or filaggrin gene mutations. However, these potential limitations seem unlikely to have accounted for the clear patterns observed in this study and we believe that the results allow inferences to be made in terms of the prevalence and trends of AD in the resource-rich populations.

## Conclusions

Determining prevalence is a crucial step in understanding the impact of AD on affected people and health systems. Our results confirm that in affluent countries one-tenth of the general population suffers from AD annually and suggest that AD prevalence has not increased over time.

## Supplementary Information

Supplementary Information 1.

Supplementary Information 2.

## Data Availability

The data extracted from included studies and used for analyses are available upon reasonable request from the authors.
