# Supplementary figures and images for: 12-month prevalence of atopic dermatitis in resource-rich countries: a systematic review and meta-analysis
Source: Sci Rep. 2022 Sep 6;12:15125. doi: 10.1038/s41598-022-19508-7 (PMC9448775; doi:10.1038/s41598-022-19508-7)

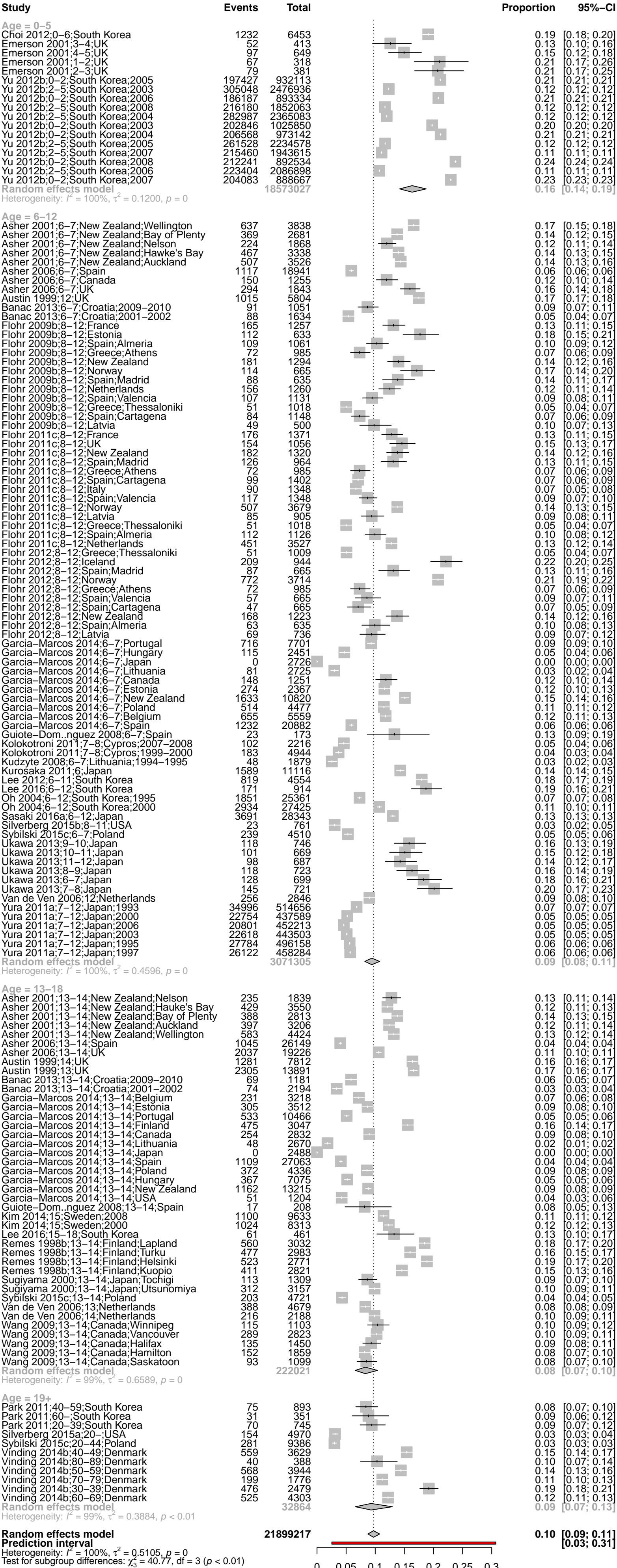

Supplement: Supplementary file 1 — Supplementary Information 1. [file 41598_2022_19508_MOESM1_ESM.pdf]
