# Supplementary material for: 12-month prevalence of atopic dermatitis in resource-rich countries: a systematic review and meta-analysis
Source: Sci Rep. 2022 Sep 6;12:15125. doi: 10.1038/s41598-022-19508-7 (PMC9448775; doi:10.1038/s41598-022-19508-7)

Supplementary Information

Scientific Reports

12-month prevalence of atopic dermatitis in resource-rich countries. A systematic review and meta-analysis

Annika Volke,[1,2]* Karolin Toompere,[3] Kaja-Triin Laisaar,[3] Marje Oona,[3] Anna Tisler,[3] Annika Johannson,[3,4] Kadi Kallavus,[3] Katrin Lang,[3] Ele Kiisk,[3] Anneli Uusküla[2,3]

[1]Department of Dermatology, Institute of Clinical Medicine, University of Tartu, Tartu, Estonia

[2]Dermatology Clinic, Tartu University Hospital, Tartu, Estonia

[3]Institute of Family Medicine and Public Health, University of Tartu, Tartu, Estonia

[4]Diagnostic Service, Pärnu Hospital, Pärnu, Estonia

***Corresponding author: Annika Volke**

Department of Dermatology, University of Tartu, Raja 31, Tartu 50417, Estonia

E-mail: annika.volke@ut.ee Phone: +372 7319702

Contents

Supplementary material 1. List of countries.

Supplementary table 1. Full electronic search strategy for OVID Medline.

Supplementary table 2. Assessments of risk of bias items by study.

Supplementary table 3. Sensitivity analysis of age-group definitions used in the AD prevalence.

Supplementary figure 1. Funnel plots for 12-month prevalence of AD across age groups.

Supplementary material 1. List of countries.

Australia, Austria, Belgium, Bulgaria, Canada, Chile, Croatia, (Republic of) Cyprus, Czech Republic, Denmark, Estonia, Finland, France, Germany, Greece, Hungary, Iceland, (Republic of) Ireland, Israel, Italy, Japan, South Korea, Latvia, Liechtenstein, Lithuania, Luxembourg, Malta, Netherlands, New Zealand, Norway, Poland, Portugal, Romania, Slovakia/Slovak Republic, Slovenia, Spain, Sweden, Switzerland, United Kingdom (of Great Britain and Northern Ireland), USA

Supplementary table 1. Full electronic search strategy for OVID Medline. Source: Ovid MEDLINE(R) 1946 to August Week 1 2016

No	Query	Results
1.	atopic dermatitis.mp. or exp Dermatitis, Atopic/	20,196
2.	atopic eczema.mp.	2,321
3.	exp Eczema/ or eczema.mp.	17,600
4.	exp Dermatitis/ or dermatitis.mp.	103,653
5.	neurodermatitis.mp. or exp Neurodermatitis/	1,685
6.	atopic skin diathesis.mp.	16
7.	1 or 2 or 3 or 4 or 5 or 6	106,474
8.	exp Prevalence/	226,438
9.	prevalence.mp.	488,147
10.	population based.mp.	87,215
11.	population-based.mp.	87,215
12.	general population.mp.	68,866
13.	8 or 9 or 10 or 11 or 12	602,267
14.	Australia.mp. or exp Australia/	134,502
15.	exp Austria/ or Austria.mp.	21,420
16.	Belgium.mp. or exp Belgium/	21,472
17.	Bulgaria.mp. or exp Bulgaria/	6,876
18.	Canada.mp. or exp Canada/	152,636
19.	chile.mp. or exp Chile/	13,432
20.	croatia.mp. or exp Croatia/	7,191
21.	czech republic.mp. or exp Czech Republic/	8,264
22.	denmark.mp. or exp Denmark/	50,447
23.	estonia.mp. or exp Estonia/	2,693
24.	Finland.mp. or exp Finland/	36,977
25.	France.mp. or exp France/	107,290
26.	greece.mp. or exp Greece/	18,934
27.	exp Hungary/ or hungary.mp.	19,769
28.	iceland.mp. or exp Iceland/	4,874
29.	exp Ireland/ or ireland.mp.	23,435
30.	israel.mp. or exp Israel/	30,382
31.	italy.mp. or exp Italy/	95,176
32.	japan.mp. or exp Japan/	150,129
33.	latvia.mp. or exp Latvia/	1,481
34.	liechtenstein.mp. or exp Liechtenstein/	212
35.	lithuania.mp. or exp Lithuania/	2,892
36.	luxembourg.mp. or exp Luxembourg/	981
37.	malta.mp. or exp Malta/	1,062
38.	exp Netherlands/ or netherlands.mp.	69,231

No	Query	Results
39.	new zealand.mp. or exp New Zealand/	55,062
40.	norway.mp. or exp Norway/	43,476
41.	exp Poland/ or poland.mp.	46,684
42.	portugal.mp. or exp Portugal/	11,999
43.	cyprus.mp. or exp Cyprus/	1,422
44.	republic of cyprus.mp.	22
45.	romania.mp. or exp Romania/	10,210
46.	slovakia.mp. or exp Slovakia/	3,567
47.	slovenia.mp. or Slovenia/	2,945
48.	spain.mp. or exp Spain/	73,389
49.	sweden.mp. or exp Sweden/	75,511
50.	switzerland.mp. or exp Switzerland/	38,778
51.	USA.mp.	67,811
52.	exp United States/ or united states of america.mp.	1,216,728
53.	eastern europe.mp. or exp Europe, Eastern/	160,721
54.	exp Europe/	1,219,077
55.	nordic countries.mp. or exp "Scandinavian and Nordic Countries"/	172,045
56.	scandinavia.mp.	3,395
57.	baltic.mp. or exp Baltic States/	8,027
58.	exp Mediterranean Region/ or mediterranean.mp.	31,052
59.	yugoslavia.mp. or exp Yugoslavia/	9,724
60.	czechoslovakia.mp. or exp Czechoslovakia/	18,660
61.	Slovak republic.mp.	513
62.	exp Korea/ or korea.mp. or exp "Republic of Korea"/	37,894
63.	US.mp.	7,792,425
64.	east* germany.mp. or exp Germany, East/	13,807
65.	exp Germany, West/ or west* germany.mp.	30,841
66.	west* europe.mp.	8,042
67.	south* europe.mp.	3,865
68.	north* europe.mp.	5,852
69.	germany.mp. or exp Germany/	165,123
70.	great britain.mp. or exp Great Britain/	330,548
71.	scotland.mp. or exp Scotland/	26,656
72.	exp England/ or england.mp.	114,925
73.	exp Wales/ or wales.mp.	30,283
74.	exp Northern Ireland/ or north* ireland.mp.	5,774
75.	united kingdom.mp.	28,670
76.	UK.mp.	73,484
77.	east* europe.mp.	6,036
78.	14 or 15 or 16 or 17 or 18 or 19 or 20 or 21 or 22 or 23 or 24 or 25 or 26 or 27 or 28 or 29 or 30 or 31 or 32 or 33 or 34 or 35 or 36 or 37 or 38 or 39 or 40 or 41 or 42 or 43 or 44 or	10,040,276

No	Query	Results
	45 or 46 or 47 or 48 or 49 or 50 or 51 or 52 or 53 or 54 or 55 or 56 or 57 or 58 or 59 or 60 or 61 or 62 or 63 or 64 or 65 or 66 or 67 or 68 or 69 or 70 or 71 or 72 or 73 or 74 or 75 or 76 or 77	
79.	7 and 13 and 78	3,730

Supplementary table 2. Assessments of risk of bias items by study.

Source	Was probablity sampling used?	Representativeness of the sample	Adequacy of response rate	Established validity of the survey instrument	Were sampling features accounted for in analysis?
Choi 2012[34]	high	unclear	low	low	low
Cibella 2011[61]	high	unclear	low	low	unclear
Duhme 1998[49]	low	low	low	low	low
Emerson 2001[54]	high	unclear	low	low	low
Flohr 2011[76]	NA	NA	NA	low	NA
Flohr 2012[75]	NA	NA	NA	low	NA
Flohr 2009[74]	NA	NA	NA	low	NA
Garcia-Marcos 2014[77]	NA	NA	NA	low	NA
Anderson 2004[55]	low	low	low	unclear	low
Grize 2006[73]	unclear	unclear	low	low	unclear
Guiote-Domínguez 2008[71]	low	low	unclear	low	low
Hong 2014[35]	high	unclear	low	low	unclear
Kudzyte 2008[66]	low	low	low	low	unclear
Kurosaka 2011[42]	high	unclear	low	low	unclear
Lee 2011[36]	low	low	unclear	low	low
Augustin 2015[50]	low	low	low	low	low
Miyake 2011[43]	low	low	high	low	low
Miyake 2004[44]	low	low	high	low	low
Mortz 2001[57]	low	low	low	low	low
Radtke 2014[51]	low	low	low	low	low
Sasaki 2016[45]	high	unclear	low	low	low
Saunes 2007[70]	low	low	low	low	low
Silverberg 2015[59]	low	low	unclear	low	low
Ukawa 2013[46]	high	unclear	unclear	low	low

Kim 2014[72]	low	low	high	low	low
Asher 2001[67]	low	low	low	low	low
Asher 2006[78]	NA	NA	NA	low	NA
Kolokotroni 2011[64]	low	low	low	unclear	low
Austin 1999[56]	low	low	low	low	low
Lee 2012[37]	low	low	unclear	unclear	unclear
Lee 2016[38]	low	low	unclear	unclear	unclear
Banac 2013[63]	low	low	unclear	unclear	low
Oh 2004[41]	low	low	unclear	unclear	unclear
Remes 1998[65]	low	low	low	low	low
Silverberg 2015[60]	low	low	low	low	low
Sugiyama 2000[47]	unclear	unclear	unclear	low	unclear
Sybilski 2015[68]	low	low	high	low	unclear
van de Ven 2006[69]	low	low	low	unclear	unclear
Wang 2010[62]	low	low	high	low	unclear
Yura 2011[48]	low	low	low	low	low
Zutavern 2005[52]	low	low	low	unclear	unclear
Worm 2006[53]	low	unclear	high	low	low
Vinding 2014[58]	low	low	high	low	low
Park 2011[39]	low	low	high	low	low
Yu 2012[40]	low	unclear	low	low	low

Abbreviations: NA – not applicable

Supplementary table 3. Sensitivity analysis of age-group definitions used in the AD prevalence.

Age group	No of studies	AD prevalence % [95% CI]
Data from studies with inconsistent age groups as a separate category		
Between age groups 6-18	25	7.58 [6.01; 9.52]
6-12	81	9.41 [8.21; 10.76]
13-18	41	8.29 [6.57; 10.40]
Data from studies with inconsistent age groups merged to the age group 6-12 years		
6-12	106	8.94 [794; 0.1005]
13-18	41	8.29 [6.57; 10.40]
Data from studies with inconsistent age groups merged to the age group 13-18 years		
6-12	81	9.41 [8.21; 10.76]
13-18	66	8.03 [6.80; 9.46]

Supplementary figure 1. Funnel plots for 12-month prevalence of AD across age groups.

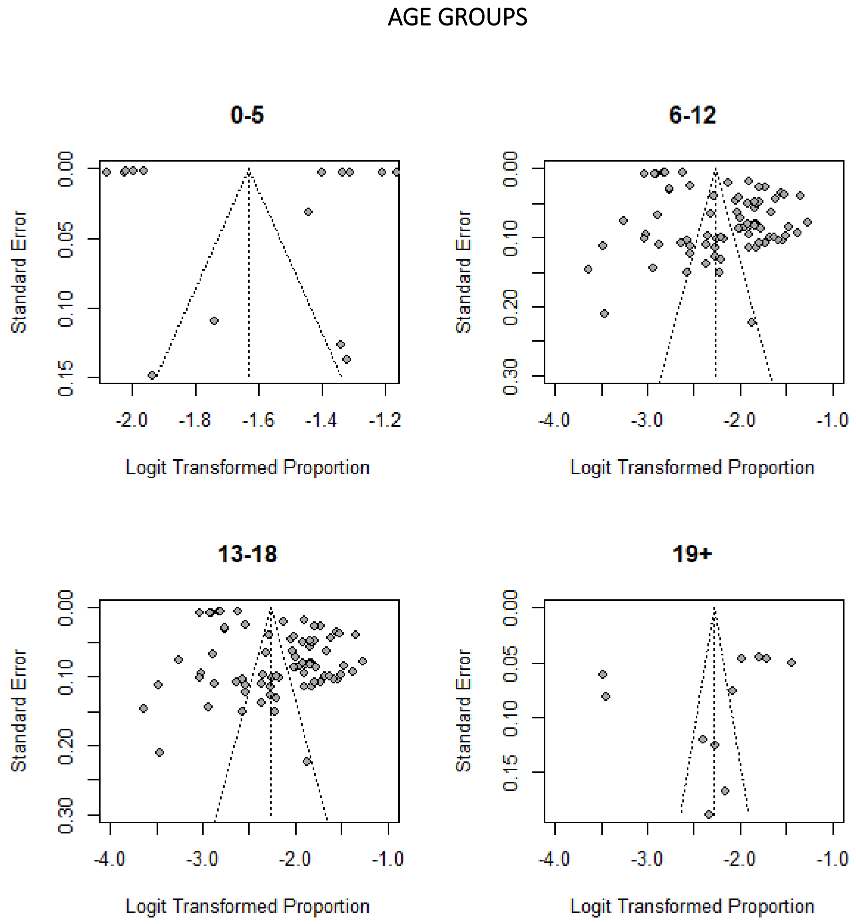

Supplement: Supplementary file 2 — Supplementary Information 2. [file 41598_2022_19508_MOESM2_ESM.pdf]
